# Impact of bottle size on in-home consumption of sugar-sweetened beverages: protocol for a feasibility and acceptability study

**DOI:** 10.1186/s40814-015-0037-8

**Published:** 2015-11-20

**Authors:** Eleni Mantzari, Gareth J. Hollands, Rachel Pechey, Susan Jebb, Theresa M. Marteau

**Affiliations:** 1 Behaviour and Health Research Unit, University of Cambridge, Cambridge, UK; 2 Nuffield Department of Primary Care Health Sciences, University of Oxford, Oxford, UK

**Keywords:** Sugar-sweetened beverages, SSBs, Consumption, Free sugars, Bottle size

## Abstract

**Background:**

Intake of free sugars in the population exceeds recommendations, with the largest source in the diet being sugar-sweetened beverages (SSBs). SSB consumption is linked to adverse health consequences and contributes to health inequalities, given greater consumption amongst the most deprived. One possible intervention is to reduce the available sizes of SSB packaging but there is an absence of evidence that this would reduce consumption. Based on evidence from studies targeting food consumption that people consume less when exposed to smaller package sizes, we hypothesise that presenting SSBs in smaller containers reduces consumption. We are planning a crossover randomised controlled trial to assess the impact of presenting a fixed volume of SSB in different bottle sizes on consumption at home. To reduce the uncertainties related to this trial, we propose a preliminary study to assess the feasibility and acceptability of the recruitment, allocation, measurement, retention and intervention procedures.

**Methods/design:**

Households which purchase at least 2 l of regular cola drinks per week and live in Cambridgeshire, UK will have a set amount of a cola SSB (based on their typical weekly purchasing of cola) delivered to their homes each week by the research team. This total amount of cola will be packaged into bottles of one of four sizes: (i) 1500 ml, (ii) 1000 ml, (iii) 500 ml or (iv) 250 ml. A crossover design will be used in which households will each receive all four of the week-long interventions (the four different bottle sizes) over time, randomised in their order of presentation. Approximately 100 eligible households will be approached to assess the proportion interested in actively participating in the study. Of those interested, 16 will be invited to continue participation.

**Discussion:**

The findings will inform the procedures for a crossover randomised controlled trial assessing the impact of presenting a fixed volume of SSB in different bottle sizes on consumption at home. The findings from such a trial are expected to provide the best estimate to date of the effect of container size on beverage consumption and inform ongoing scientific and policy discussions about the effectiveness of this intervention at reducing population intake of free sugars in beverages.

**Trial registration:**

ISRCTN14964130

## Lay summary

What is known already

- The consumption of sugar-sweetened beverages is associated with adverse health consequences, including obesity, metabolic syndrome, diabetes and poor oral health, and health inequalities, given higher consumption amongst those who are more socially deprived.
- One possible intervention to reduce sugar-sweetened beverage consumption is to reduce the container sizes (i.e. bottles and cans) in which these drinks are presented. However, evidence regarding the effectiveness of this intervention is currently lacking.

What this study will add

- The findings of this feasibility study will provide the information needed to finalise the design of a planned crossover randomised controlled trial assessing the impact of bottle size on in-home consumption of sugar-sweetened beverages.

Policy implications

- The findings of the planned trial will inform policy concerning the use of sizing interventions to reduce consumption of sugar-sweetened beverages to improve population health.

## Background

There is increasing concern that the consumption of free sugars (i.e. all mono- and disaccharides added to foods by the manufacturer, cook or consumer, plus sugars naturally present in honey, syrup and fruit juices [1]) can have serious adverse health consequences. Specifically, the intake of such sugars at high levels leads to weight gain and an increased risk of non-communicable diseases (NCDs) [2] and the development of dental diseases, particularly, dental caries [3, 4]. Such concerns have led the World Health Organization (WHO) to advise limiting the consumption of free sugars to less than 10 % of total daily caloric intake, with reductions below 5 % highlighted as having additional health benefits [5]. However, global intake of free sugars exceeds these recommendations, with the largest source in the diet being sugar-sweetened beverages (SSBs) [6, 7]. SSBs are consumed widely around the world, including in the UK and USA, with carbonated drinks being the predominant product. For example, in 2013, carbonated drinks were purchased by 91 % of households in the UK and USA [8, 9]. Also in 2013, the average consumption of carbonated drinks per person in the UK was 103 l [8] and approximately 170 l in the USA [10].

SSBs contain free sugars, including sucrose, high fructose corn syrup and other energy-containing sweeteners. A 330-ml portion of a carbonated SSB typically contains approximately 35 g (i.e. seven teaspoons) of sugar and provides approximately 140 cal, generally without any nutritional value [11, 12]. In recent decades, consumption of SSBs has increased globally [13]. Given that consumers do not tend to reduce their energy intake to compensate for the additional energy provided by SSBs [14], SSB consumption increases total daily energy intake [15–17], has thus been linked to weight gain and the development of obesity [18–20], metabolic syndrome and diabetes [18, 19, 21], as well as hypertension [22], dental diseases [23], gout [24, 25], non-alcoholic steatohepatitis [26], and is associated with an estimated 180,000 global deaths per year [27]. SSB intake may also contribute to observed inequalities in health outcomes, as it is socially patterned: In high-income countries, heavy consumption and purchasing are more common amongst adults and children of lower socio-economic status [28–31]. In the UK, for example, approximately 25 % of the population of regular SSB consumers live in the most deprived areas (i.e. areas ranked in the most deprived quintile based on their Index of Multiple Deprivation (IMD) scores [32]) compared to 15 % who live in the least deprived areas (i.e. areas ranked in the least deprived quintile based on their IMD scores) [29].

Given the contribution of free sugars, especially from SSBs, to the rise in chronic disease and to health inequalities, curbing their intake has been identified for public health action [5, 33]. Identifying effective interventions is recognised as key to this action. Reducing the size of containers in which SSBs are available is one possible intervention. In the USA, a recent attempt to regulate the size of products in order to reduce their consumption comprised a ban on the sale of sugary drinks larger than 16 oz (473 ml) in many out-of-home settings [34]. The proposal has yet to be approved. In the UK, there are recent examples of companies reducing the portion sizes of sugary drinks as part of their voluntary pledges under the government’s Public Health Responsibility Deal in England (https://responsibilitydeal.dh.gov.uk/about/). This intervention, increasing the availability of smaller portion sizes, has not been evaluated for its impact on beverage consumption or purchasing. In theory, several, often conflicting, mechanisms have been suggested as underlying the impact of the size of products, including beverages, on consumption [35, 36]. One possibility is that people are guided by available external cues and so perceive the amount served to them as representing an appropriate portion size (a norm). They therefore consume less when offered smaller portions and more when offered larger portions [36]. Perception of appropriate portion sizes might in turn be influenced by individuals’ personal and social norms about what constitutes a suitable amount to consume. As larger portions have become more prevalent and normalised, smaller portions might be considered less appropriate [37] and therefore fail to reduce consumption. Contrary to this is the suggestion that smaller sizes might reduce consumption, regardless of whether they are considered a suitable portion, by making additional consumption more effortful [38] or due to people’s tendency to consume a specific number of product units in any one episode of consumption regardless of the unit size (referred to as the ‘unit bias heuristic’) [39].

From the above, it is clear that the theoretical literature on this issue is complex and does not allow for confident predictions to be made regarding the impact of bottle size on the consumption of SSBs. However, a large number of studies have examined the effects of portion and package sizing on the consumption of foodstuffs (such as cereal and snack foods) [35–37], and a recent Cochrane review on this topic found that people consume less food the smaller the package size to which they are exposed [38, 39]. It is unclear whether such findings are generalizable to beverage consumption, as there are no studies, to our knowledge, that examine the effects of manipulating the package size of drinks. The lack of relevant studies is also confirmed by the Cochrane review [38, 39]. Based on the aforementioned finding, however, we hypothesise that presenting SSBs in smaller containers will reduce consumption.

To assess this hypothesis and address the absence of relevant evidence, we are proposing a crossover randomised controlled trial to assess the impact of presenting a fixed volume of sugar-sweetened beverages in different bottle sizes on consumption in homes. The focus of this study is on the size of bottles containing cola, given that cola is the most consumed carbonated drink in the UK [8] and is available in a wide range of container sizes. However, prior to conducting this trial, there is a need to reduce key uncertainties related to its design, including the feasibility and acceptability of delivering the intervention to households and replacing existing SSBs and the feasibility of assessing consumption. There are also uncertainties relating to the specific bottle sizes the trial should focus on. As such, we are initially proposing a preliminary study with the aim of assessing the feasibility and acceptability of the procedures for recruitment, allocation, measurement, retention and intervention delivery of the aforementioned randomised controlled trial.

### Aim and objectives

The aim of the current study is to assess the feasibility and acceptability of conducting a randomised controlled trial of the impact of bottle size on consumption of cola in homes.

The specific objectives of the current study are to describe and assess the following:

i. Feasibility of recruiting participants from eligible households into the trial, including estimating the recruitment rate
ii. Characteristics of recruited households, to judge the likelihood of recruiting a sample varied in potential effect modifiers (i.e. socioeconomic factors)
iii. Bottle sizes that are most appropriate for use in the planned trial
iv. Feasibility and practicalities associated with delivering the intervention
v. Acceptability of the intervention
vi. Awareness of the purpose of the intervention
vii. Possible effect size, to inform sample size calculations for the planned trial
viii. Feasibility and acceptability of the assessment procedures
ix. Feasibility of collecting end - point data
x. Loss-to-follow up rates

## Methods/design

### Setting

The proposed feasibility and acceptability study will be conducted in a community setting comprising residential households, located in Cambridgeshire, UK, including Cambridge City, South Cambridgeshire and East Cambridgeshire, which have a total population of 363,800.

### Design

**Fig. 1:**
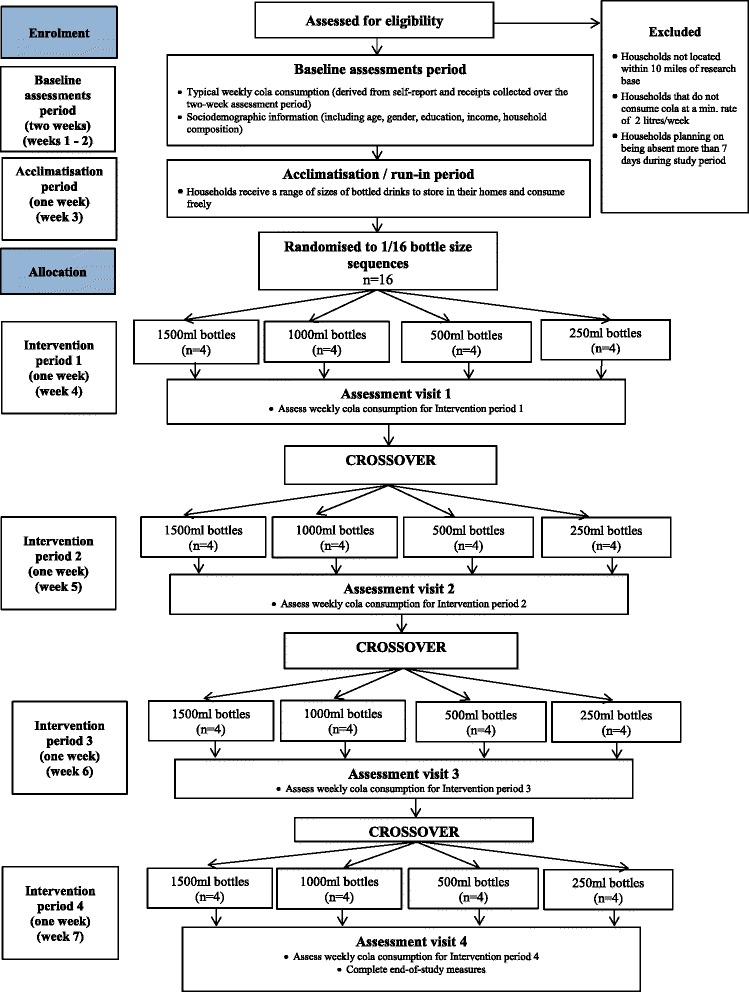
Flow of participants through the study

We propose to use a crossover design in which general population households will each be exposed to four intervention conditions over time, randomised in their order of presentation. The unit of randomisation is the household. The four conditions will comprise receipt of a given quantity of a carbonated cola sugar-sweetened beverage (SSB), sub-divided into bottles of one of four different sizes (Fig. 1).

A within-subjects, crossover design is being used because of the variability between households, which will vary considerably by size, as well as their social and psychological characteristics. Being able to control for these differences makes a within-subjects crossover design preferable to a between-subjects design. It also maximises statistical power thereby increasing efficiency of research resource in terms of accruing the greatest reduction in uncertainty possible from participating households.

### Participants

Participating households will be of any size or composition. We define households as people who live together, who may or may not be related but who share all or most drink and food within the house. This includes households consisting of

- Single members
- Couples (married or cohabitating)
- Families with school age children
- Single parents with dependent children
- Families with extended family members living in the house (e.g. grandparents)

In line with the characteristics of the population of SSB consumers in the UK [29], 50 % of the recruited households will be from areas of high deprivation, as defined by their Index of Multiple Deprivation (IMD)^1^ [32] score, with areas falling under the fourth and fifth IMD quintile considered highly deprived. Inclusion criteria for participating households are as follows:

i. Purchase regular (not low sugar) Coca-Cola**®** or Pepsi Cola**®** at a minimum rate of 2 l a week.
ii. Reside in or near to Cambridge, UK, within 10 miles of the research team base.
iii. Do not plan to be away from home for longer than 7 days during the study period.

We will recruit one individual from each eligible household to act as a household representative, who will consent to participation in the study for the entire household and provide all necessary data. In order to evaluate recruitment rates, we will initially approach 100 eligible households with the aim of assessing how many would be interested in taking part in the study and how many would complete the run-in period (see ‘Procedure’ section for details). We assume that households completing this period would be likely to continue, completing at least one intervention period. Of the households completing the run-in period and expressing a willingness to continue with the study, we will invite 16 to continue their participation and undergo the intervention periods. If fewer than 16 households complete the run-in period, we will approach additional households.

Households will be identified and recruited through a research agency.

### Intervention

The four conditions will comprise receipt of a given quantity of a carbonated cola sugar-sweetened beverage (SSB), sub-divided into bottles of one of four different sizes:

i. 1500 ml bottles,
ii. 1000 ml bottles,
iii. 500 ml bottles and
iv. 250 ml bottles.

In any 1-week, households will receive their preference of cola (Coca-Cola**®** or Pepsi Cola**®**) in just one size of bottle, with the number of bottles being determined by the total volume of cola each household receives, which will be fixed across all four intervention periods. This fixed volume will be determined with reference to the volume of cola households purchased during a 2-week baseline period, as assessed by till receipts and by self-report, rounded up to the nearest multiple of 3 l. For example, if households purchased 2 l per week, they will receive 3 l during each intervention period; if they purchased three and a half litres, they will receive 6 l etc. Households purchasing amounts already consisting of a multiple of 3 l will receive this exact amount (e.g. if they purchased 3 l, they will receive 3 l). Volumes consisting of multiples of 3 l are needed to avoid the total quantity varying systematically between intervention periods, thereby confounding the effect of altering the bottle size with volume. During each intervention week, households will be given the opportunity to receive additional deliveries should they want these.

All participating households will receive all interventions according to a pre-specified random order (see ‘Randomisation’ section for details). During the first intervention period, four households will receive cola in each of the intervention bottle sizes, subsequently crossing-over to the other conditions during the remaining three intervention periods, until all households have been exposed to all conditions. All intervention periods will be conducted during school term times, in order to avoid increased consumption during any specific period due to the increased presence of children in the homes. During the intervention periods, a researcher will visit participating households to deliver the total volume of the cola (Coca-Cola**®** or Pepsi Cola**®** depending on their preference) SSB for the forthcoming week. The researcher will remove any existing SSB products that are in the household, with financial compensation provided for any removals. At the end of each week, households will be requested to pay for the SSB drinks they consume at their usual purchasing rate. During the intervention periods, households will be requested to continue to keep all bottles whether the contents are consumed, partially consumed or not consumed. They will be told that the reason for this is to accurately estimate how much they need to pay for their drinks, based on the amount they consumed.

### Randomisation

Randomisation will be restricted to ensure that (a) equal numbers of households receive each of the four bottles sizes at each intervention period (Table 1) and (b) each bottle size is equally likely to be preceded and followed by each other bottle size. These conditions are imposed to ensure that the order in which the interventions are presented is counterbalanced and that outcomes are not affected by potential order and carry-over effects. In a larger trial, such as the future one we are planning, we do not foresee the need for imposing such restrictions, as randomisation of a large number of households is expected to minimise the chance of imbalances in the order of the interventions.

**Table 1:** Number of households allocated to each bottle size during each intervention period

	Bottle size			
	250 ml	500 ml	1000 ml	1500 ml
Intervention period 1	*n* = 4	*n* = 4	*n* = 4	*n* = 4
Intervention period 2	*n* = 4	*n* = 4	*n* = 4	*n* = 4
Intervention period 3	*n* = 4	*n* = 4	*n* = 4	*n* = 4
Intervention period 4	*n* = 4	*n* = 4	*n* = 4	*n* = 4

We will initially randomly select four sequences (out of 24 available sequences) dictating the order in which households receive each bottle size. These four sequences will then be used to determine the other 12 sequences needed to balance the initial allocations to ensure that the aforementioned conditions are met. Each household will be randomly allocated to one of these 16 sequences. In this manner, the order in which each household receives each bottle size will be pre-specified at the start of the study. The randomisations will be determined during the run-in period (see ‘Procedure’ section) by a statistician independent from the research team, with the assistance of computer software.

### Procedure

Participation will comprise seven stages:

1. Two-week recruitment and baseline assessment period (see ‘Baseline assessments’ section for details).
2. One-week run-in period. Households will receive a range of differently sized bottled drinks to store in their homes to consume freely. This period functions to acquaint them with both the idea that a wide range of bottle sizes is available and that drinks will be delivered to them over the course of the study. More generally, it will build credibility of the study as a consumer research exercise. Study randomisation will be conducted during this period.
3. First intervention period: 1 week in duration.
4. Second intervention period: 1 week in duration.
5. Third intervention period: 1 week in duration.
6. Fourth intervention period: 1 week in duration.
7. End of study and debriefing.

Stages 1–2 will be completed by any number of eligible households out of the 100 approached that agree to do so.

Stages 3–7 will be completed by 16 households, randomly selected from those completing stages 1–2, who also express a willingness to continue participation in the study. If only 16 households agree to continue their participation, all will be invited to complete these additional stages.

Each household will receive £150 worth of shopping vouchers at the end of the study for completion of all intervention periods and follow-up assessments. This amount was determined based on the national minimum wage rates per hour and the level of commitment and amount of time spent participating in the study (approximately 25 h during 5-week period). Households completing the run-in period but not invited to continue their participation, or not interested in continuing, will receive £30 worth of shopping vouchers.

Households will not be fully informed at recruitment of the purpose of the interventions and of the study’s aim, as it is assumed that such knowledge could potentially influence the outcome. Instead, household representatives will be told that the study involves a consumer research exercise aiming to determine whether and how different bottles affect people’s consumption experiences. Specifically, they will be told that the study will explore whether different bottle sizes influence perceptions of taste, level of enjoyment and satisfaction associated with beverage consumption, perceived product quality and likelihood that the product will be purchased in the future, as well as attitudes towards different bottles, including their appeal and user - friendliness.

### Baseline assessments

At enrolment, household representatives (i.e. individuals who are recruited from each household to provide the necessary data) will be asked to give written informed consent on behalf of their household for participating in the study and for adhering to the study procedures. Following this, they will be requested to complete a questionnaire to record their household’s demographic characteristics, including the number of adults and children living in their home, their age, gender, highest educational qualification and annual household income. They will also be asked to indicate how much cola per week their household usually buys for in-home consumption, as well as the total amount their household drinks outside the home. The amount of cola typically consumed per week by each household will also be estimated based on grocery shopping till receipts, which household representatives will be asked to collect during a 2-week period. Discrepancies between amounts indicated by till receipts and self-report will be discussed with household representatives, in order to determine the household’s typical weekly consumption as accurately as possible.

### Follow-up assessments

At the end of each of the intervention weeks, household representatives will be asked to estimate the amount of SSB their household consumed in the preceding week and to indicate whether any SSB was consumed by non-household members (i.e. visitors). To build credibility for the cover story they will also be asked to rate their consumption experiences, as well as the experience of any visitors they might have had, who consumed part of their SSB stock.

In order to determine whether households believed the cover story or were aware of the purpose of the intervention and of the study’s aim, at the final follow-up assessment, they will be requested to state what they thought the study was about. Awareness of the study’s aim will also be assessed during interviews with household representatives, which will be conducted after the end of all intervention periods. Household representatives will be fully debriefed on the study aims at the end of this final assessment session. The debriefing process will include an explanation of the study’s scientific aim and the reasons for not revealing this at recruitment (i.e. that awareness of the intervention’s purpose was expected to influence the outcome), as well as information regarding the adverse consequences of excessive SSB consumption. This information will be provided both verbally by a member of the research team, as well as in writing. At this point, household representatives will be given the opportunity to make any enquiries regarding the study and address any related issues they might have. They will also be asked to provide written consent for their household’s collected data to be used having been informed of the scientific aims of the study.

### Outcomes and measures

Feasibility outcomes

- Recruitment rates
- Number of households discontinuing participation at follow-ups
- Awareness of the study aim, assessed through (i) questionnaire and (ii) qualitative interviews
- Practical problems associated with
  - ◦The randomisation procedure
  - ◦Delivering the intervention
  - ◦Collection of consumption-related data

Acceptability outcomes assessed through qualitative interviews

- Acceptability of the
  - ◦Interventions
  - ◦Study procedures
  - ◦Assessment procedures

Other outcomes:

- Characteristics of participating households, assessed through questionnaire
  - ◦Index of Multiple Deprivation scores (derived from postcodes)
  - ◦Total household income
  - ◦Household composition (number of adults; number of children)
  - ◦Highest education qualification obtained by any person within the household
  - ◦Gender of all household members
  - ◦Age of all household members
- Volume of cola in millilitres consumed by the household during each of the week-long intervention periods, measured
  i. Objectively, by recording the numbers of empty and remaining full bottles. The remaining volume of partly consumed bottles will be weighted and converted to millilitres.
  ii. Subjectively, through self-report via questionnaire.

### Sample size

This study is designed as a feasibility and acceptability study to inform a future, large-scale trial. Consequently, a formal power calculation is not required [7, 40]. The number of eligible households to be approached was selected as a means to facilitate estimation of recruitment and active participation rates. The number of households invited to participate in the intervention periods was guided by the need to ensure that equal numbers of households receive each of the four bottles sizes at each intervention period (i.e. the need to recruit a multiple of four households). The specific sample sizes were selected predominantly based on available resources (i.e. staff and funding) and our previous experiences of reducing key uncertainties in feasibility studies prior to finalising designs for randomised controlled evaluations of complex behavioural interventions.

### Qualitative component

Household representatives will be interviewed at the end of the study in order to assess acceptability of the intervention and of the study procedures, including the removal of existing drinks. The interviews will also be used to explore whether participants were conscious of the study’s primary aim and if so, whether they thought this knowledge influenced their household’s consumption of the cola. Interviews will be semi-structured and last approximately 1 h. They will be recorded and sent for external transcription. Transcripts of the interviews will be anonymised.

### Data analysis

The main analysis of this study will include descriptive statistics of feasibility and acceptability outcomes, including recruitment and attrition rates. We will also calculate the average change from baseline in SSB consumption during each intervention period to estimate possible effect sizes from which to power the main trial.

Analysis of the anonymised data gathered through the semi-structured interviews will be conducted following the principles of the Framework method [41].

### Research governance

The study is funded by a grant from the Department of Health Policy Research Program (Policy Research Unit in Behaviour and Health [PR-UN-0409-10109]). The Department of Health has no role in the study design, data collection or analysis, decision to publish or preparation of the manuscript. Ethical approval was obtained by the University of Cambridge Psychology Department Research Ethics Committee (reference number Pre.2015.20). Management, data storage and analysis will be conducted at the Behaviour and Health Research Unit, Primary Care Unit, Department of Public Health and Primary Care, University of Cambridge.

## Discussion

Recent years have witnessed a marked awareness of the many adverse health consequences of high intake of free sugars, especially in the form of SSBs [18–27, 42, 43]. Curbing SSB consumption, which has increased rapidly around the world in the last decades [13], has accordingly become a public health priority [11, 33], with recent research focusing on identifying effective interventions.

A number of interventions aiming to reduce SSB consumption exist and have been assessed in recent studies. These include the use of education [44] and the provision of caloric information through labelling [45–47]. However, the effects of these have been, at best, modest with the latter being observed in one study to increase, rather than decrease, consumption [47]. To achieve significant reductions in SSB consumption, it has been argued that regulatory approached are needed [48], such as increases in the price of SSBs through taxation [13, 49]. Although there is some evidence to suggest that in principle, an SSB tax could reduce consumption [50], further evidence is needed based on observed rather than modelled effects [51]. Preliminary results of the effects of an SSB tax in Mexico (constituting approximately a 10 % increase in price per litre) shows a 10 % decline in purchases in the first quarter of 2014 compared to the first quarter of 2013 [52]. The policy awaits further evaluation. Discussions regarding implementation of taxes on SSBs in other countries have been met with objections, such that it would unfairly penalise low-income individuals and households and would unfairly single out one type of food [51]. This highlights the need to also explore further, potentially more acceptable ways of reducing SSB consumption. Another potentially effective regulatory approach [34] that might be more acceptable than taxation is to restrict serving sizes. However, the impact of such an intervention has yet to be thoroughly examined. Simulation studies suggest that the introduction of a restriction on available beverage sizes, such as the ban proposed by the mayor of New York City on sales of SSBs larger than 16 oz (473 ml), could have favourable effects on consumption [53, 54]. However, there is currently a lack of experimental evidence—as opposed to evidence from simulation studies—to support the use of such an intervention. In addition, the impact of presenting the same total amount of a beverage in multiple, smaller packages on SSB consumption is unknown.

The current feasibility and acceptability study is designed to finalise the design and conduct of a future, full-scale trial to assess whether reducing the size of the bottles in which SSBs are presented can reduce consumption. Its primary purpose is to address key design uncertainties for the trial, including the feasibility of recruiting eligible households, the practicalities of delivering the intervention to households and replacing existing SSBs and the feasibility of assessing consumption, as well as the specific bottle sizes that should be targeted. It will be the first study to our knowledge to explore the use a package-size intervention for reducing SSB consumption. Its robust experimental design and naturalistic setting are expected to enhance the value of the findings, whilst inclusion of a qualitative component will also provide a detailed understanding of the acceptability of the intervention. It should be noted,however, that as this is a feasibility study, findings will require replication in larger and more representative samples.

The success of this feasibility and acceptability study will be judged on a number of criteria, including (i) our target sample size is met by approaching 100 households, (ii) recruited households match the deprivation level of typical SSB consumers in the UK (i.e. 50 % are from areas falling under the fourth and fifth IMD quintile), (iii) the majority of participants are not aware of the study’s scientific aim and (iv) at least 12 households out of 16 complete the first intervention period. Should some of these indicators of success not be met, we will consider changes to the study design and procedures before deciding whether and how to proceed with the trial. Specifically, we will consider additional recruitment methods and perhaps widen the target recruitment areas and/or modify the specified eligibility criteria. Larger than expected attrition rates will be accounted for in the trial sample size calculations. Knowledge of the study’s scientific aim could affect the results. Therefore, if we find that most participants are aware of the study aims, we will consider an alternative cover story.

In conclusion, evidence regarding the effectiveness of sizing interventions for reducing SSB consumption is currently lacking. The proposed study will provide information needed to finalise the design of a future crossover randomised controlled trial assessing the impact of reducing bottle size on in-home consumption of SSBs. This trial will provide the best estimate to date of the potential effect of altering the packaging size of SSBs on consumption and thereby inform policy concerning the use of such interventions to reduce consumption to improve population health and reduce health inequalities.

## Footnotes

1 Residential postcodes will be used to calculate IMD scores. The IMD is a measure of deprivation in England based on area of residence. It measures deprivation at the small area level, that is, the Lower Layer Super Output Area. It is derived from seven indices of deprivation, including income, employment, health deprivation and disability, education skills and training, barriers to housing and services, crime and the living environment. These are combined into a single deprivation score for each small area in England.
